# Pelvic nerve endometriosis: MRI features and key findings for surgical decision

**DOI:** 10.1186/s13244-025-02005-6

**Published:** 2025-06-19

**Authors:** Justine Bourg, Edouard Ruaux, Pierre Adrien Bolze, Marie Gavrel, Mathilde Charlot, François Golfier, Isabelle Thomassin-Naggara, Pascal Rousset

**Affiliations:** 1 Department of Radiology, Hospices Civils de Lyon, Lyon Sud University Hospital, Lyon 1 Claude Bernard University, Pierre Bénite, France; 2 Department of Gynecology and Obstetrics, Hospices Civils de Lyon, Lyon Sud University Hospital, Lyon 1 Claude Bernard University, EMR 3738, Pierre Bénite, France; 3 Department of Radiology, Service Imageries Radiologiques et Interventionnelles Spécialisées, Hôpital Tenon, Assistance Publique Hôpitaux de Paris, Sorbonne Université, Paris, France

**Keywords:** Endometriosis, Magnetic resonance imaging, Pelvic plexus, Pelvic pain

## Abstract

**Abstract:**

Endometriosis is a prevalent gynecological disorder in women of reproductive age. It is the leading cause of chronic pelvic pain. While the mechanisms underlying this pain remain elusive, rare cases of pelvic nerve involvement can result in severe, debilitating symptoms, adding complexity to the clinical landscape. Nerve involvement typically results from the direct extension of deep infiltrating endometriosis, though it may also occur in isolation. The nerves most commonly affected include the inferior hypogastric and lumbosacral plexuses, as well as the sciatic, pudendal, obturator, and femoral nerves. Early and accurate diagnosis is essential for the effective management of the pain and the prevention of irreversible nerve damage. Given the limitations of transvaginal ultrasonography in visualizing the lateral compartment, MRI is considered the gold standard for detecting and evaluating pelvic nerve involvement. Through the use of optimized protocols to enhance the visualization of nerves and their anatomical landmarks, radiologists play a key role in the identification of endometriotic lesions. A comprehensive and structured radiology report is essential for surgical planning, as nerve involvement often requires precise interventions to alleviate symptoms and restore quality of life.

**Critical relevance statement:**

Accurate identification and a structured reporting of pelvic nerve endometriosis in the lateral compartment are pivotal to guide surgical decision-making and optimize patient outcomes.

**Key Points:**

- Pelvic nerve endometriosis is often overlooked, underestimated by clinicians, and underdiagnosed on imaging.
- Timely nerve involvement diagnosis prevents permanent damage in pelvic pain with neurological symptoms.
- Deep endometriosis in the lateral compartment may extend to the pelvic nerves.
- The inferior hypogastric plexus, sacral plexus, sciatic, and pudendal nerves are commonly affected.
- A dedicated MRI protocol with 3D T2-weighted sequence ensures accurate pelvic nerve assessment.

**Graphical Abstract:**

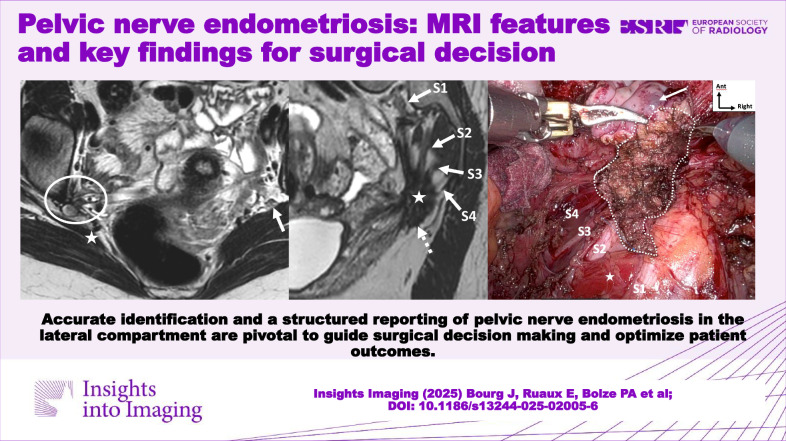

## Introduction

Endometriosis is a chronic inflammatory condition characterized by the presence of endometrial-like tissue outside the uterine cavity. It affects approximately 10% of women of reproductive age [1]. Endometriosis can be divided into three phenotypes: ovarian endometrioma, superficial endometriosis, and deep infiltrating endometriosis [2]. Deep endometriosis (DE) is defined by the infiltration of endometrial-like tissue beneath the peritoneal surface. It is usually nodular, invasive to adjacent structures, and associated with fibrosis and disruption of normal anatomy [3]. It typically involves the uterosacral ligaments, torus uterinum, vagina, rectosigmoid, rectovaginal septum and urinary tract. In rare cases, endometriosis may involve pelvic nerves, with the inferior hypogastric plexus, sacral plexus, and sciatic nerve being most frequently affected [4]. The true incidence of neural endometriosis remains uncertain, but is likely underestimated, potentially due to low clinical suspicion or limited familiarity with neural anatomy among abdominal or pelvic radiologists. In addition, diagnosis may be difficult in cases of isolated pelvic endometriosis, without concomitant endometriosis elsewhere in the pelvis [5].

The clinical presentation of neural endometriosis is variable and often nonspecific, including symptoms such as pain, weakness, numbness, bladder or rectal dysfunction, and lower limb dysfunction in case of severe lesions [6]. Symptoms can either be cyclic or constant. When endometriosis is not already diagnosed, these symptoms are rarely attributed to this disease at first, but rather to rheumatologic, osteoarticular, or neurological causes. This misdirection contributes to significant delays in diagnosis, as initial explorations often rely on non-specialized imaging techniques or protocols. Additionally, musculoskeletal radiologists tend to be less familiar with endometriosis imaging.

Early diagnosis is crucial to effectively manage the patient’s pain and prevent irreversible nerve damage. While ultrasonography is the first-line imaging modality for assessing deep pelvic endometriosis, it has consistent limitations due to its restricted field of view, particularly in lateral compartments—its limited contrast resolution in subperitoneal space, and its operator dependence [7]. In this context, magnetic resonance imaging (MRI) is considered the imaging modality of choice. It allows assessment and detailed mapping of the extent and severity of neural endometriosis, which can alter the medical and surgical management of patients [8].

The management of these patients is inherently complex and typically requires a multidisciplinary approach to determine the optimal treatment strategy. It often involves a combination of hormonal therapy, surgery, and pain management. When surgery is decided upon, laparoscopic surgery—frequently assisted by robotic technology—is preferred for cases involving sacral nerve roots and pelvic nerves [9].

This article provides a comprehensive review of the pathophysiology of neural endometriosis, proposes an optimized MRI protocol tailored for the evaluation of pelvic nerves, and highlights key anatomical landmarks crucial for the accurate detection and analysis of each nerve. Finally, it outlines the key MRI features of endometriotic neural involvement that should be reported for effective surgical planning.

## Physiopathology of neural endometriosis

DE is strongly associated with pelvic pain, severe dysmenorrhea, and deep dyspareunia [10]. Among women with chronic pelvic pain, the prevalence of endometriosis is estimated at approximately 70% [11]. This persistent pain is often the most debilitating symptom, significantly impacting women’s quality of life. Depending on the role of the nerve fibers, somatic and vegetative symptoms such as pain, weakness, numbness, bladder and rectal dysfunction may occur [12]. Since pelvic nerve endometriosis is a rare clinical presentation, it is often overlooked by practitioners. The cyclical nature of catamenial symptoms, when present, can be a critical diagnostic clue and should alert the practitioner.

However, the mechanisms underlying endometriosis-associated pain remain poorly understood. Nerve fibers are believed to play a central role in both the initiation and modulation of pelvic pain in women with endometriosis. Increasing evidence suggests that endometriotic lesions exhibit heightened neural density [13]. Through this process known as neuroangiogenesis, ectopic endometriotic implants establish their own distinct neural and vascular networks [13]. These lesions engage in bidirectional interactions with sensory, sympathetic, and parasympathetic fibers, promoting inflammation, angiogenesis, proliferation, and further innervation. This crosswalk may contribute to the development of the disease. It is thought to affect neuronal activity in the central nervous system, increasing pain perception [14].

Additionally, ectopic endometrial cells may directly invade or irritate peripheral nerves, contributing to pain [10]. However, the retroperitoneal course of pelvic nerves raises questions about the unclear pathogenesis of endometriosis. Sampson’s theory of retrograde menstruation suggests that menstrual reflux allows endometrial cells to implant on peritoneal surfaces with peritoneal diverticula facilitating their migration into adjacent nerves [15, 16]. Pelvic peritoneal pockets—either formed secondarily through endometriosis-induced inflammation and scarring or existing as primary retraction defects—may serve as reservoirs for ectopic tissue. These defects are commonly found in areas such as the pouch of Douglas or posterior ovarian fossa, with a potential extension towards retroperitoneal tissues like the sciatic notch [17]. A notable right-sided predominance of sciatic nerve involvement may be attributed to the peritoneal fluid flow and the anatomical location of the sigmoid colon, which limit cell reflux into the left posterior hemipelvis pockets, thus protecting the left lumbosacral plexus and the sciatic nerve [18].

However, this theory does not explain cases of distant extrapelvic endometriosis or those without peritoneal involvement. In such cases, the perineural spread theory, proposed by Possover et al and Siquera et al, offers an alternative explanation, supported by MRI and surgical evidence [19, 20]. This theory suggests that endometriosis can disseminate along pelvic autonomic nerves—similar to the perineural spread observed in pelvic malignancies—extending from the uterine plexus to the lumbosacral plexus. As no single theory can explain all the manifestations of endometriosis, multiple mechanisms are likely at play. Hypotheses such as hematogenous or lymphatic spread, coelomic metaplasia, and embryonic cell rest, are also considered [4, 21].

Either way, neural endometriosis can compress, distort, or infiltrate the epineural tissue, possibly leading to nerve entrapment and fibrosis. In addition, local edema around DE lesions may contribute to symptoms even in the absence of direct neural infiltration [22].

## Treatment principles

Regardless of the route of pelvic nerve involvement in endometriosis, early diagnosis and treatment of neural endometriosis are crucial to prevent irreversible nerve damage caused by chronic inflammation, fibrosis, and adhesions. Management is based on a multidisciplinary approach, integrating clinical symptoms and MRI features. Medical management is based on GnRH analogs, analgesics, tricyclic antidepressants, and antiepileptics [23]. When conservative treatments fail to relieve pain symptoms and impact patients’ quality of life, surgery becomes the method of choice. The laparoscopic retroperitoneal approach with nerve-sparing technique and somatic nerve decompression is considered the gold standard for appropriate surgical radicality [24]. Possover et al introduced the concept of laparoscopic neuronavigation, a parasympathetic nerve-sparing technique based on nerve electrostimulation [25].

Finally, pelvic pain management must be comprehensive, addressing the multifactorial and often interrelated factors of pain. Endometriosis is particularly associated with myofascial pain, due to active myofascial trigger points, which can amplify and perpetuate chronic pain [26].

## MRI protocol

### Standard optimized MRI protocol

MRI is the reference technique for diagnosing neural endometriosis and offers a comprehensive roadmap for assessing DE, which is crucial for preoperative decision making. The MRI protocol should follow the European Society of Urogenital Radiology and the Society of Abdominal Radiology guidelines [27, 28]. Imaging can be performed on a 1.5- or 3-Tesla MRI system, though 3T-MRI may present limitations such as increased specific absorption rate and susceptibility artifacts [29]. Both societies recommend moderate bladder distension and the administration of an anti-peristaltic agent to minimize bowel motion artefacts. The core protocol should include T2-weighted (T2W) MRI sequences in two or three planes and T1-weighted (T1W) MRI. However, conventional sequences may be insufficient for detecting pelvic nerve involvement due to their long course and small caliber. In this context, 3D sequences with multiplanar reformats are valuable for their high spatial resolution. The use of 3D-T2W MRI sequences is increasingly common for the evaluation of deep pelvic endometriosis, particularly for improved assessment of the lateral compartments [30]. These sequences also enhance visualization of nerves, which often have an oblique course.

The 3D DIXON technique is currently the reference standard for T1W imaging. Non-fat-suppressed T1W MR images allow clear identification of nerves surrounded by hyperintense perineural fat, and help assess their anatomical relationships. In contrast, fat-suppressed T1W MR images are essential for detecting T1W-hyperintense hemorrhagic cystic foci/lesions surrounding or within the nerve.

Although gadolinium injection is not routinely recommended for the evaluation of pelvic endometriosis, it may be beneficial when pelvic nerve involvement is suspected [22]. Contrast-enhanced T1W fat-suppressed MRI can facilitate the detection of DE surrounding the pelvic nerve. However, the extent of the lesion may be overestimated due to perilesional inflammation.

### Advanced MRI protocol

In addition to this optimized MRI protocol, other techniques may be considered for more advanced analysis. MR neurography can be used to identify pelvic nerve abnormalities caused by endometriosis [31]. It is based on high T2W imaging with fat suppression and vascular suppression (mainly veins) to enhance nerve signal intensity, which is clearly visible due to endoneurial fluid. Both non-contrast techniques and gadolinium-based contrast agents can be used to suppress the vascular signal, with gadolinium reducing the T1 relaxation time of blood to match that of fat tissues [32]. Multiplanar reconstruction and maximum intensity projection techniques aid in detecting focal nerve abnormalities. This sequence, which adds approximately 8 min to the imaging time, can be an optional add-on sequence when neural endometriosis is clinically suspected or requires re-evaluation [22]. It provides a detailed 3D view which may be helpful in procedure planning, but can also help to better visualize and track the involved nerve, assess its degree of interruption, and help to better see muscle atrophy, if present. Muscle evaluation should focus on size asymmetry—larger, edematous muscles indicate acute denervation, while smaller, fatty muscles indicate chronic denervation atrophy [33].

Diffusion tensor imaging (DTI) is an advanced MRI technique that assesses microstructural nerve abnormalities. It primarily assesses the integrity of fiber tracts, measuring fractional anisotropy, an index of the architectural organization of tissue [34]. Tractography can also provide neural tracts on three-dimensional (3D) images. Although clinical research is limited, DTI seems to be a promising technique to evaluate nerve abnormalities in endometriosis; lower fractional anisotropy values have been found in women with endometriosis compared to healthy controls, suggesting microarchitectural abnormalities in the affected sacral roots [35, 36].

## MRI features and key findings for pelvic nerve involvement to report in endometriosis

**Fig. 1:**
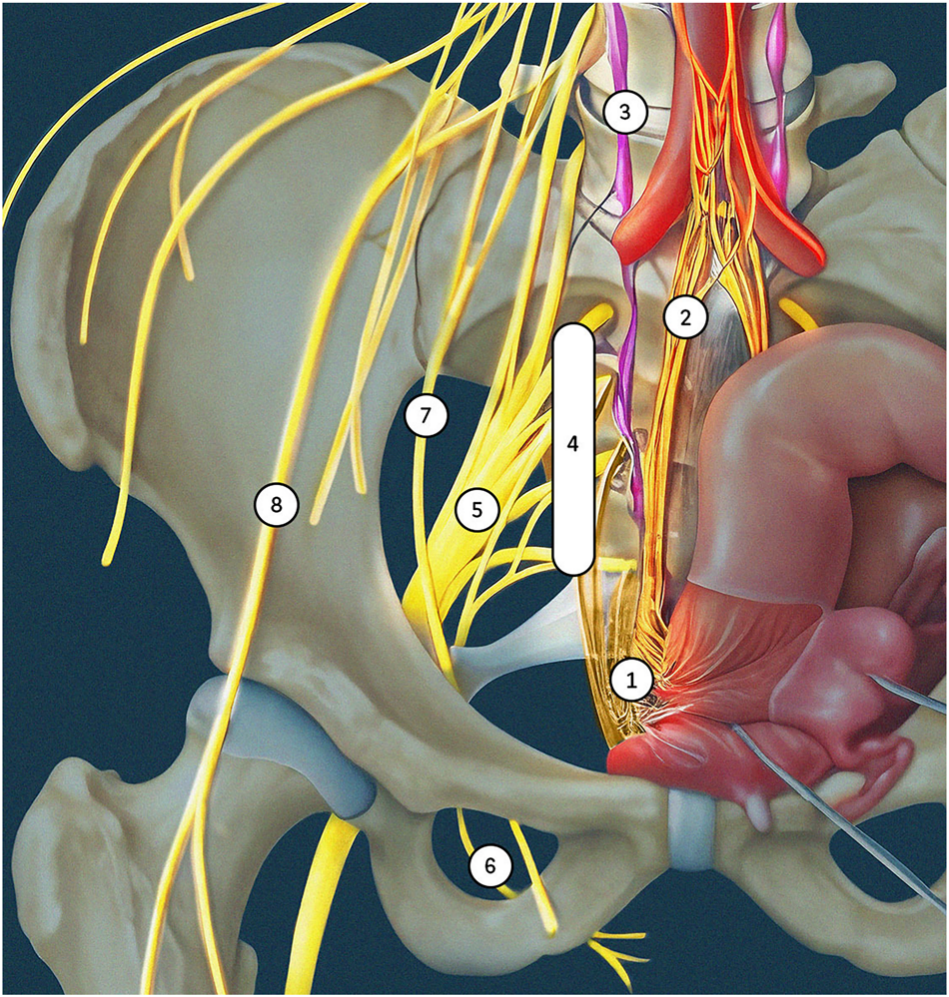
Anatomical illustration of somatic and autonomic pelvic nerves in a female pelvis (frontal view, adapted from Alkatout et al [37]). 1: inferior hypogastric plexus, 2: hypogastric nerve, 3: sympathetic trunk with chain of ganglia, 4: sacral roots (S1–S4), 5: sciatic nerve, 6: pudendal nerve, 7: obturator nerve, and 8: femoral nerve

Accurate identification and a structured reporting of pelvic nerve endometriosis in the lateral compartments are crucial for guiding surgical decision making and optimizing patient outcomes (Table 1 and Fig. 1) [37].

**Table 1:** Pelvic nerve endometriosis: anatomical landmarks, MRI features, and key points for surgical planning

	KEY ANATOMICAL LANDMARKS	MRI FEATURES	KEY POINTS FOR SURGICAL PLANNING
INFERIOR HYPOGASTRIC PLEXUS	Mediolateral parametrium Posterolateral parametrium containing the sacrorectal septum: lateral to the mesorectal fascia and beneath the uterosacral ligament and the ureter.	Fibrotic nodular infiltration, more or less spiculated, with the loss of the normal aspect of the subperitoneal paracervical cellulovascular tissue and the pararectal cellular layer	Relationships of the endometriosis lesion with: - Ureter and uterine artery - Sacral roots - Levator ani muscle - Pelvic fascia, piriformis muscle - Gluteal and internal iliac vessels - Sciatic nerve
SACRAL ROOTS	- Anterior to the piriformis muscle - Indentation within the muscle for S2 and S3	Fibrotic thickening of the sacral roots (mostly S3 and S4) in continuity with the infiltration of the posterolateral parametrium	- Extension along the nerve root up to the foramen - Relationship with the presacral fascia
SCIATIC NERVE	- Anterior to the piriformis muscle - Greater sciatic notch, above and outside the ischial spine	Endometriotic infiltration extending from the ovarian fossa towards the sciatic notch, or in a more isolated manner, without other pelvic identifiable endometriosis lesion Thickening with the loss of “spaghetti-like” appearance of the sciatic nerve, often with hemorrhagic cystic foci	- Size of the lesion - Extrapelvic extension through the greater sciatic foramen - Intrinsic or extrinsic involvement - Relationship to the obturator nerve and the inferior gluteal vessels - Muscle denervation
PUDENDAL NERVE	- Origin: anterior to mid-course of the piriformis muscle - Middle course: ischial spine, between the sacrotuberous and sacrospinous ligaments, then entering Alcock’s canal - Terminal course: ischiorectal fossa at the end of Alcock’s canal, following the pudendal vascular pedicle	At three different levels: - Proximal nerve involvement in continuity with the infiltration of posterolateral parametrium and S3/S4 nerves - Fibrotic thickening at the entrance of Alcock’s canal - Fibrotic thickening in the perineal region adjacent to the episiotomy scar	Relationships of the endometriosis lesion with: - Levator ani muscle - Obturator internus muscle - Vagina and anal canal (in case of extensive lesion in the perineal region)
OBTURATOR NERVE (Rare)	- Medial border of the psoas muscle - Pelvic brim - Obturator foramen	Extension of a large endometriosis lesion from the ovarian fossa going through the mediolateral parametrium up to the obturator nerve Retractile fibrotic infiltration with possible hemorrhagic cystic areas along the pelvic wall, tracking the course of the nerve	Relationships of the endometriosis lesion with: - Ureter - Obturator internus muscle - Muscle denervation
FEMORAL NERVE (Rare)	- Lateral border of the psoas muscle - Between the psoas and iliacus muscles - Under the inguinal ligament, following the round ligament in the canal	Infiltrating retractile tissue either in the region iliopsoas muscle or in the inguinal region (adjacent to the distal round ligament)	Relationships of the endometriosis lesion with the: - Inguinal canal - Common femoral artery and vein

Neural endometriosis lesions typically appear on MRI similarly to other DE lesions, presenting as T2-hypointense solid nodules or fibrotic thickenings. These lesions, may also show microcystic or hemorrhagic foci, indicative of active ectopic glandular tissue, and often involve the pelvic nerve, which may appear thickened, interrupted, or encased.

### Inferior hypogastric plexus

The inferior hypogastric plexus is the most commonly affected structure within the lateral compartments in endometriosis [38]. This complex neural network provides autonomic innervation of the pelvic viscera. It is formed by the convergence of the hypogastric nerves (which arise from the superior hypogastric plexus), the pelvic splanchnic nerves (which arise from the anterior branches of the sacral roots S2–S4) and the sacral splanchnic nerves (which arise from the sacral sympathetic trunk at the S2 and S3 ganglia) [39]. The inferior hypogastric plexus branches into two main pathways: the anterolateral branch, which innervates the uterus and lower bladder, and a posteromedial branch, which targets the posterolateral aspect of the rectum and gives rise to the inferior rectal plexus [39].

The inferior hypogastric plexus spans from anterior to posterior through the lateral compartments, within the mediolateral and posterolateral parametrium (Supplemental Fig. 1) [40, 41]. It is located below the ureter and deep uterine vein, both of which serve as important landmarks during surgery [39]. It is typically characterized by a roughly triangular configuration with a posterior base (Supplemental Fig. 2) [40]. The sympathetic contingent (from the hypogastric nerves and S2–S3 ganglia) relaxes the detrusor muscle, contracts the internal urethral and anal sphincters, promoting continence. In contrast, the parasympathetic contingent (from the pelvic splanchnic nerves) stimulates the detrusor contraction, facilitating bladder emptying, and modulates the enteric nervous system of the left colon [40].

**Fig. 2:**
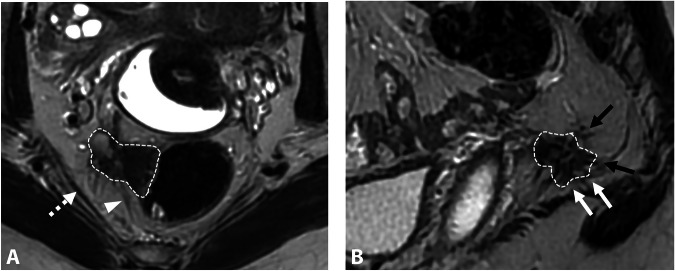
DE in a 29-year-old woman with dysmenorrhea, deep dyspareunia, and dyschezia. **A** Axial and (**B**) sagittal T2W MR images show a right subperitoneal infiltrative lesion (dotted lines) involving the anterolateral rectal wall and extending to the mesorectal fascia (**A**, white arrowhead) and beyond into the right posterolateral parametrium. The infiltration of the inferior hypogastric plexus shows spiculated margins (**B**, black arrows) but no extension to the sacral roots or the pelvic wall (**A**, dashed arrow). Note the safety fat line between the lesion and the iliococcygeus muscle of the levator ani muscle (**B**, white arrows). Laparoscopic surgery confirms the involvement of the inferior hypogastric plexus and a cleavage plane with the levator ani muscle and sacral roots

Thus, inferior hypogastric plexus involvement usually presents with vegetative symptoms such as bladder or rectal dysfunction (e.g., dysuria, bladder fullness, dyschezia) and vaginal dryness. Sometimes, catamenial sciatica occurs due to traction on the sacral roots via connecting splanchnic fibers. The plexus itself is not visible on MRI due to its complex, spiderweb-like structure. Therefore, understanding its relationship with the pelvic viscera and key anatomical landmarks is crucial. MRI typically shows infiltration from a large retrocervical lesion (torus uterinum and the proximal and/or distal uterosacral ligaments) to the sacrorectal septum of the posterolateral parametrium, with possible associated involvement of the mediolateral parametrium [42]. These lesions are often severe, with involvement of the rectal wall [43]. On MRI, they present as fibrotic nodular infiltration with low signal intensity on T2W, and loss of the normal aspect of the subperitoneal pararectal cellular sheet beneath the uterosacral ligament level and lateral to the mesorectal fascia [38]. Spiculated margins, particularly visible in the sagittal plane, allow assessment of the posterior extension to the sacral roots and deep extension to the iliococcygeus muscle of the levator ani (Fig. 2) [38]. Preoperative MRI is crucial for predicting postoperative complications, as excision of these lesions can lead to urinary and rectal dysfunctions, often due to damage to the inferior hypogastric plexus [44]. The MRI report should specify the distance and relationship of the endometriosis lesion to the ureter and uterine artery anterolaterally, to the sacral roots posteriorly, to the levator ani muscle inferiorly and the parietal pelvic fascia laterally, extending to the piriformis muscle and the branches of the gluteal and/or internal iliac vessels [38]. Extension to the sciatic nerve is rare.

### Sacral plexus

The sacral plexus, formed by the L4–S4 ventral rami, runs along the anterior surface of the piriformis muscle and serves as a significant anatomical landmark [45]. It provides motor and sensory innervation to the pelvis and the lower limbs.

On MRI, the sacral roots, although decreasing in caliber from S1 to S4, are well traced at the level of the foramen and at least in their proximal course (Supplemental Figs. 3 and 4). They extend inferiorly, above, through, or below the piriformis muscle and then towards the greater ischial notch, except for S4. The S2 and S3 nerve roots pass through the piriformis muscle in most cases [46], with an indentation within the muscle, which facilitates their visualization. However, in the distal course, the S3 and S4 roots are not or barely visible.

The symptomatology is polymorphic and depends on the affected sacral roots. It may include pudendal and gluteal pain, sciatica, pelvic organ dysfunctions such as bladder hyperactivity, urinary urgency, constipation, and dyschezia [47].

Sacral plexus involvement typically results from direct infiltration by a large retrocervical lesion with posterior extension to the inferior hypogastric plexus and then through the connecting pelvic splanchnic nerves.

**Fig. 3:**
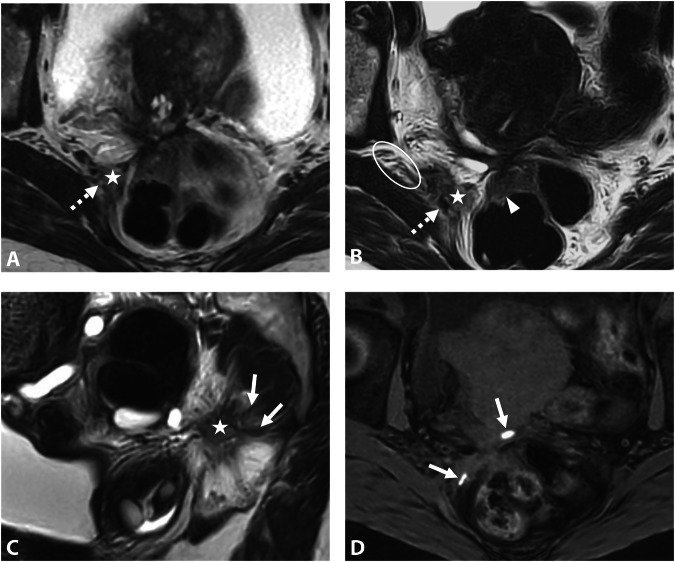
DE in a 42-year-old woman, with a history of rectal shaving 10 years ago, who presented with a recurrence of symptoms, in particular deep dyspareunia and right sciatica. **A**, **B** Axial T2 and (**C**) sagittal T2W MR images show right subperitoneal infiltrative lesion involving the anterolateral rectal wall (**B**, arrowhead), extending to the right posterolateral parametrium with inferior hypogastric plexus involvement (stars), up to the pelvic wall with encasement of hypogastric vessels (**A**, **B**, dashed arrows) and contact with the piriformis muscle. The posterior spiculated margins come into contact with the S3 and S4 sacral roots (**C**, arrows) and the posterior part of the right sciatic nerve (**B**, circle). **D** Axial fat-suppressed T1W MR image reveals T1-hyperintense endometriotic hemorrhagic microcysts (arrows). The surgical procedure confirmed extrinsic involvement and included a shaving of the right sacral roots and right sciatic nerve, as well as a section of the hypogastric vessels

**Fig. 4:**
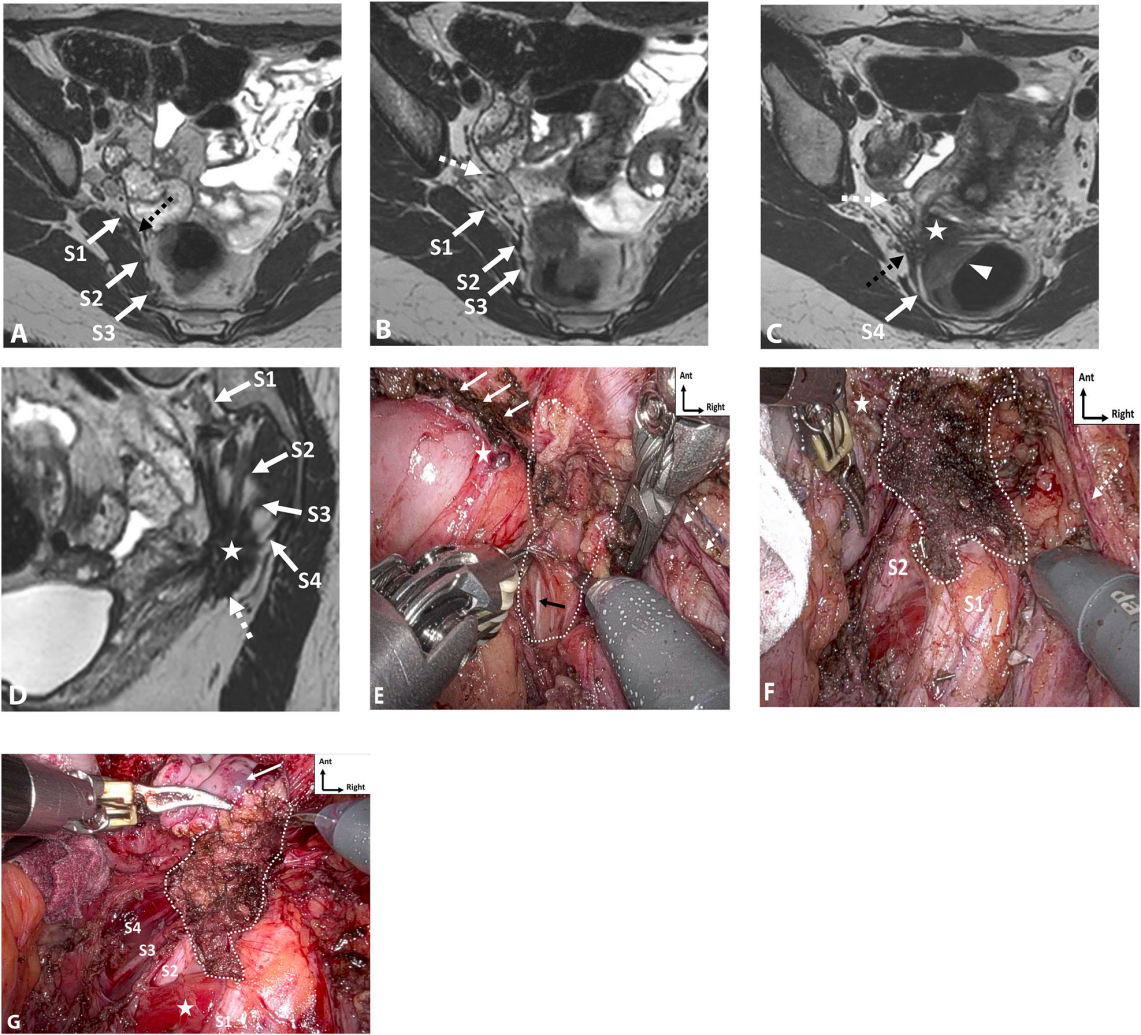
Severe endometriosis in a 31-year-old woman with dysmenorrhea, dyspareunia, and dysesthesia in the right lower extremity. **A**–**C** Axial 3D T2W images from top to bottom show endometriotic infiltration of the rectal wall (**C**, arrowhead) and posterior vaginal fornix (**C**, star), with complete extension to the right posterolateral parametrium including the inferior hypogastric plexus (black dashed arrows) with posterior attraction of S1 and circumferential involvement of S2, S3 and S4. Note the absence of extension of the ureter (**B**, **C**, white dashed arrow), the piriformis muscle, or internal iliac vessels. **D** Sagittal 3D reconstruction T2W image at the level of the right posterolateral parametrium showing involvement of the inferior hypogastric plexus (star) extending inferiorly to the levator ani muscle (dashed arrow), and posteriorly to sacral roots S2, S3, and S4, and with spiculation up to S1 but no involvement. **E** The robotic laparoscopic view shows a complete adhesion (white arrows) between the anterior rectal wall (star), the uterus and vagina, the peritoneal infiltration is being subperitoneal (dotted line). Note the hypogastric nerve (black arrow) passing through the inferior hypogastric plexus affected by the endometriosis and the course of the ureter (dashed arrows) distant from the endometriotic lesion. **F** The robotic laparoscopic photography view shows, after careful dissection, the upper part of the endometriotic lesion (dotted line) involving the rectal wall (star), S2, and partially attracting S1. Note the course of the ureter (dashed arrow). **G** Robotic laparoscopic view showing, after dissection of the endometriotic lesion (dotted line) from the rectal wall, involvement of the posterior vaginal fornix with a submucous hemorrhagic cystic component (arrow) after colpotomy, of S2, S3, and S4 and retraction of S1. Note the absence of involvement of the piriformis muscle (star). A complete nerve-sparing resection was performed, with S1 being retracted but removable from the lesion, and S2–S4 being circumscribed but without macroscopic intrinsic infiltration

On MRI, sacral roots may appear thickened with fibrotic infiltration, extending continuously with the sacrorectal septum infiltration. It mainly affects the roots S3 and S4 due to their lower, more posterior course (Fig. 3), whereas S1 and S2 are generally located above the endometriotic extension (Fig. 4) [47]. While surgical findings often reveal endometriotic traction or extrinsic involvement, accurately assessing the degree of infiltration on MRI remains challenging due to the small caliber of these sacral roots [12].

Rarely, neural extension may progress along the sacral roots to the foramen, which can be better visualized on multiplanar sagittal plane reconstruction, especially in cases of hemorrhagic implants (Supplemental Fig. 5). In such cases, the lesion’s relationship with the foramen and the presacral fascia should be reported.

### Sciatic nerve

The sciatic nerve, the largest peripheral nerve in the body, arises from the convergence of the ventral roots of L4–S3, anterior to the piriformis muscle. It exits the pelvis through the greater sciatic foramen, curves posteriorly above and out of the ischial spine, and runs laterally along to the common hamstring tendon between the ischial tuberosity and the greater trochanter [45]. It supplies motor innervation to the posterior thigh muscles and sensory innervation to the lower limb, except for the medial part.

On MRI, the sciatic nerve is clearly visible on both sides of the greater sciatic notch, with a “spaghetti-like” appearance. While its course is well traced in the axial plane, the coronal plane appears to be more relevant for detailed assessment (Supplemental Fig. 6).

The hallmark symptom is a cyclic sciatica associated with menstruation, and progressively shorter pain-free intervals. Patients usually report posterior thigh pain radiating down to the limb and the foot, sometimes accompanied by muscle weakness (foot drop), sensory loss, and reflex alterations [48].

Sciatic nerve involvement is typically identified at the sciatic notch [22]. It may result from endometriotic infiltration extending from the ovarian fossa towards the sciatic notch, or in a more isolated manner, without other identifiable pelvic endometriotic lesions. Isolated cases could be explained by the presence of a peritoneal diverticulum (pocket sign) [49] or the theory of perineural spread, especially in the absence of other endometriosis lesions.

**Fig. 5:**
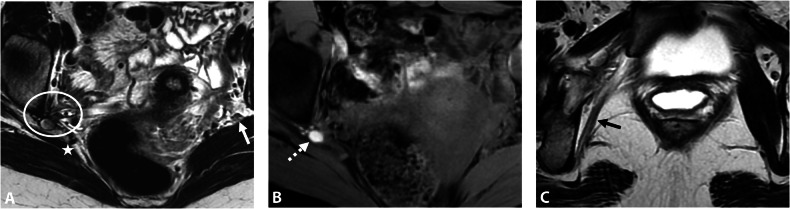
DE in a 31-year-old woman with right catamenial sciatica. **A** Axial T2W MR image shows fibrotic thickening in the right sciatic notch involving the right sciatic nerve (circle) with loss of its spaghetti aspect compared to the left sciatic nerve (arrow). This lesion is isolated without involvement of the ovarian fossa or the sacrorectal septum, and could illustrate the potential existence of a peritoneal diverticulum. Note the hypertrophy of the piriformis muscle (star). **B** Axial fat-suppressed T1W MR image shows diffuse T1-hyperintense endometriotic hemorrhagic microcysts (dashed arrow). **C** Axial T2W MR image shows fatty atrophy of the right obturator internus muscle (black arrow), indicating a concomitant involvement of the nerve to the obturator internus muscle passing through the sciatic notch. The patient was medically treated with an LHRH analog

MRI typically reveals fibrotic infiltration of the sciatic nerve, often with hemorrhagic cystic foci, leading to thickening and loss of its characteristic “spaghetti-like” appearance. In cases of chronic nerve involvement, fatty muscle atrophy may be observed, affecting muscles innervated by the sciatic nerve, such as those of the posterior thigh, leg, and foot [6, 50]. Atrophy also may affect the glutal and piriformis muscles, which are innervated by the posterior branches of the sacral plexus and have a close anatomical relationship to the sciatic nerve at the level of the greater sciatic notch. Similarly, the internal obturator muscle, innervated by the anterior branches of the sacral plexus, may be involved (Fig. 5). Muscle atrophy is mainly non-reversible and has a significant impact on the functional prognosis [51].

**Fig. 6:**
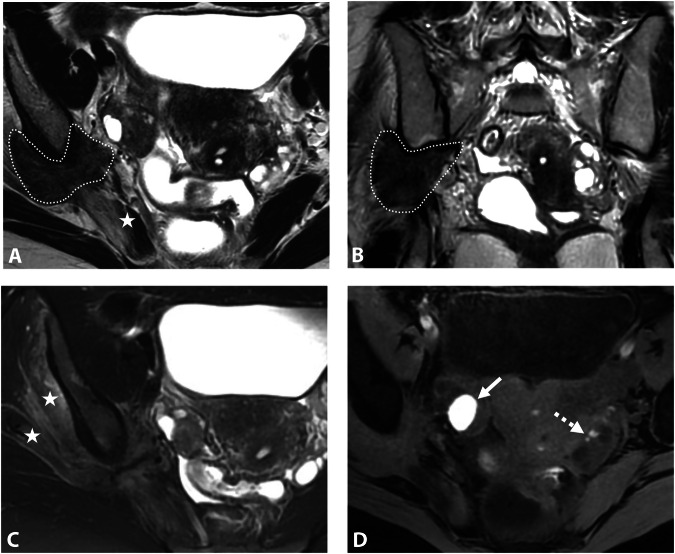
DE in a 31-year-old woman with right sciatica initially cyclical, then became chronic with walking difficulties. **A** Axial and (**B**) coronal T2W MR images show an infiltrative endometriotic mass of 5 cm (measure not shown) (dotted line) centered on the right sciatic notch with involvement of the sciatic nerve. Note the hypertrophy of the piriformis muscle (**A**, star). **C** Axial fat-suppressed T2W MR image shows denervation of the gluteus medius and minimus muscles (stars), indicating the involvement of the superior gluteal nerve in the sciatic notch, as well as the piriformis muscle. **D** Axial fat-suppressed T1W MR image shows a right-sided endometrioma (arrow) and hemorrhagic implants of the left ovary (dashed arrow). Surgical intervention was not possible due to the intrinsic involvement of the sciatic nerve, which could risk nerve damage. The patient was treated medically with an LHRH analog

Assessing intrinsic or extrinsic nerve involvement remains challenging on MRI. However, intraneural endometriosis may be suspected when the sciatic nerve appears abnormally thickened and hyperintense on T2W MR images, with visible interruption and fiber discontinuity (Fig. 6) [50].

In the preoperative assessment, it is crucial to report the lesion’s size, its relationship to the obturator nerve and the inferior gluteal vessels, and most importantly, its relationship to the greater sciatic notch and any extra-pelvic extension through the greater sciatic foramen. Indeed such cases, a combined laparoscopic and transgluteal approach may be necessary to achieve complete excision of the lesion and adequate neurolysis (or decompression) of the sciatic nerve [52].

In most cases, the nerves are embedded but not infiltrated within the epineurium, and complete release results in significant or complete relief of pain and motor issues [53]. However, if the lesion infiltrates the nerves within the epineurium, excision may involve the nerve itself, thereby increasing the complexity of surgery. Patients are more likely to experience neuropathic pain and sensorimotor disorders after surgery. Most patients with isolated sciatic nerve endometriosis who present motor symptoms, such as foot drop, experience little to no significant improvement [9, 53].

### Pudendal nerve

The pudendal nerve is formed by the ventral rami of the S2-S4 nerve roots. It has a short intrapelvic course and exits the pelvis through the greater sciatic foramen, between the piriformis muscle and the ischio-coccygeal ligament. It then curves medially along and under the ischial spine before re-entering the pelvis through the lesser sciatic foramen. It joins the internal pudendal vessels and runs along the lateral ischiorectal fossa within the pudendal (Alcock’s) canal, bounded by the obturator fascia [45]. The pudendal nerve provides motor innervation to the levator ani muscle, clitoral muscles, external anal and urethral sphincters, as well as sensory innervation of the perineum and anus [54].

Even with 3DT2 sequences, the proximal pudendal nerve is not easily visualized. It is necessary to identify anatomical landmarks of its course in order to accurately diagnose its involvement. Namely, the nerve emerges at the junction of the middle and distal third of the piriformis muscle, then passes through an anatomical window between the sacrotuberous and sacrospinous ligaments. In Alcock’s canal, the pudendal vascular pedicle serves as the landmark (Supplemental Fig. 7).

Pudendalgia presents as perineal pain or burning sensation, often worsened by sitting. Other symptoms, such as dyspareunia, dyschezia, or dysuria, are also commonly reported.

The pudendal nerve is primarily involved at three different levels.

**Fig. 7:**
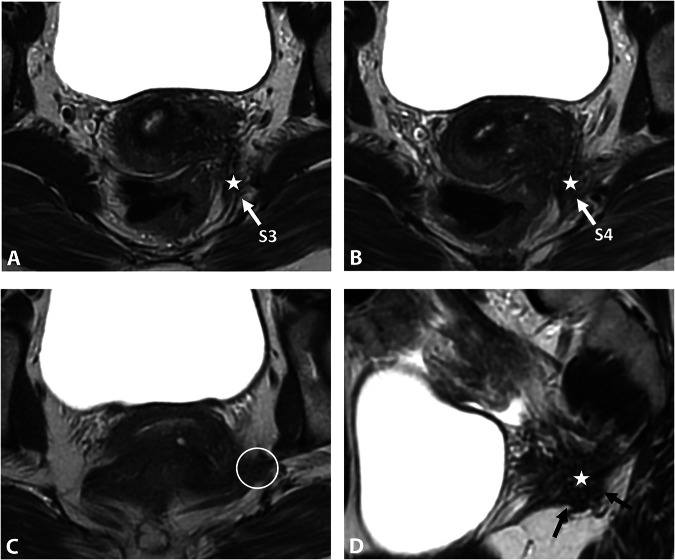
DE in a 29-year-old woman with deep dyspareunia and left pudendalgia. **A**–**C** Axial and (**D**) sagittal T2W MR images show fibrotic nodular infiltration of the left posterolateral parametrium (stars) extending to the pelvic wall and to the iliococcygeus muscle of the levator ani muscle (**D**, black arrows), involving the left inferior hypogastric plexus, the left sacral nerves courses S3 and S4, and the level of origin of the left proximal pudendal nerve (**C**, circle). The patient was treated medically with hormone therapy

At the level of the piriformis muscle, proximal pudendal nerve involvement may result from infiltration of the inferior hypogastric plexus, with posterior extension to the S3 and/or S4 roots (Fig. 7).

**Fig. 8:**
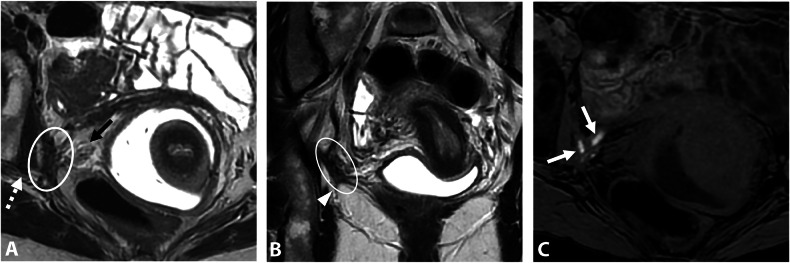
DE in a 43-year-old woman with dyspareunia and perineal pain. **A** Axial and (**B**) coronal T2W MR images show an endometriotic lesion (circle) of the right distal mediolateral parametrium extending to the pelvic wall in contact with the pudendal vascular-nerve bundle at the level of arcus tendineus of the levator ani muscle (**B**, arrowhead). The right ureter passes quite widely inside this lesion (**A**, arrow). This lesion is located in front of the right ischial spine mark (**A**, dashed arrow) and the sciatic nerve. This lesion is isolated without involvement of the ovarian fossa or the posterolateral parametrium. **C** Axial fat-suppressed T1W MR image showing diffuse T1-hyperintense endometriotic hemorrhagic microcysts (arrows). The patient was treated medically with hormone therapy

At the level of the pelvic wall near the ischial spine, at the entrance of Alcock’s canal, mid-course of the pudendal nerve involvement is often more isolated. This may be associated with a peritoneal diverticulum, similar to sciatic nerve involvement (Fig. 8) [55]. It is essential to describe the lesion’s relationship to the pudendal vascular pedicle, the sciatic nerve, and the obturator internus muscle sling posterior to the ischial spine.

**Fig. 9:**
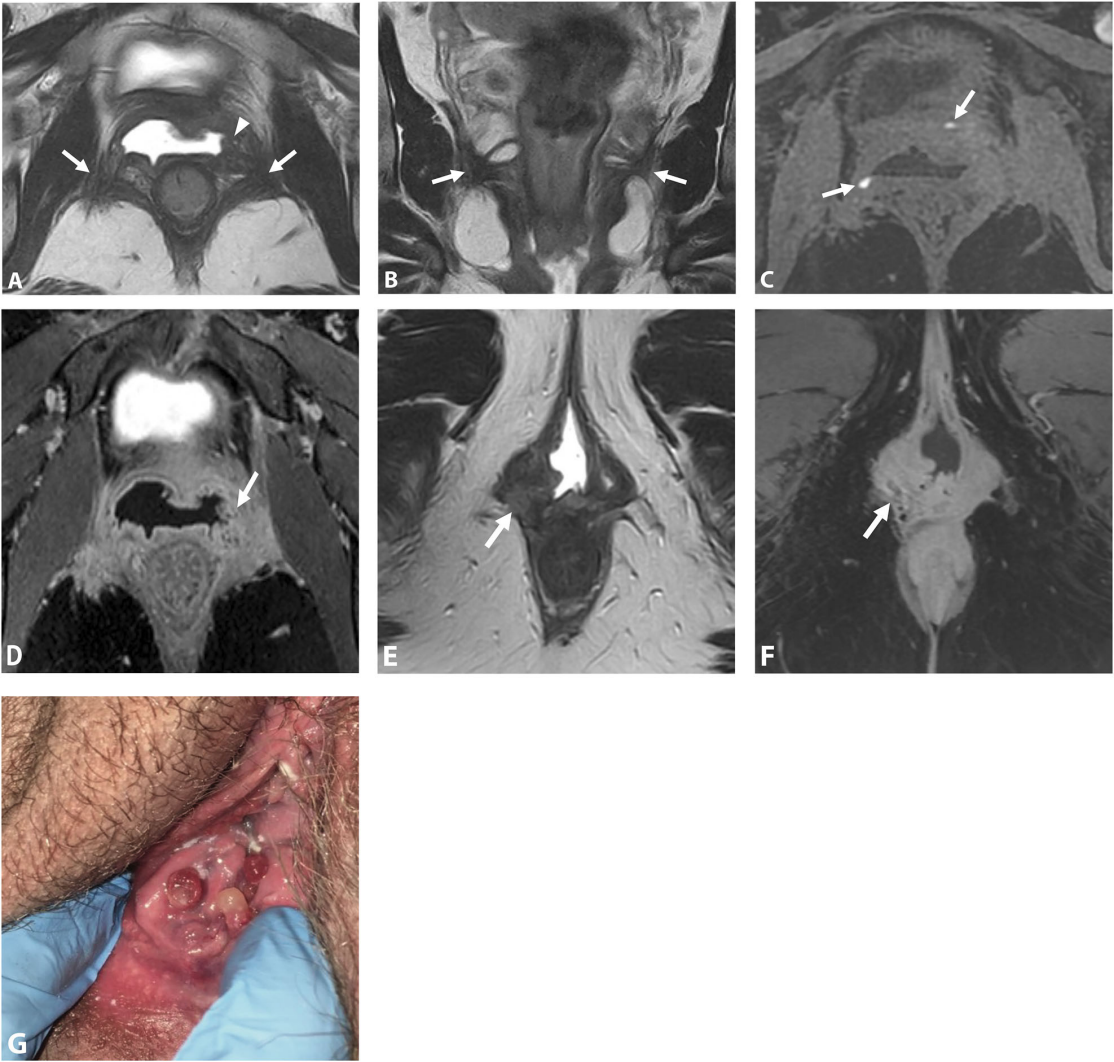
DE in a 34-year-old woman with pudendalgia after vaginal delivery with forceps extraction and bilateral mediolateral episiotomy. **A** Axial and (**B**) coronal T2W MR images show a bilateral fibrotic infiltration (arrows) of the levator ani muscle at the level of the distal course of bilateral pudendal nerves, as well as infiltration of the left part of the vagina (A, arrowhead). **C** Axial fat-suppressed T1W MR image reveals some T1-hyperintense endometriotic hemorrhagic microcysts (arrows). **D** Axial T1W post-contrast MR image shows a better delineation of the left vaginal (sub) mucosal involvement (arrow). **E** Axial T2W and (**F**) axial fat-suppressed T1W post-contrast MR images show a right vulvar lesion (arrows) correlating with clinical photographs (**G**). The patient was treated medically with hormone therapy

At the level of the perineal region, pudendal nerve involvement may result from endometriosis of an episiotomy scar extending into the ischiorectal fossa (Fig. 9) [56]. MRI can assess DE pattern in this region and evaluate the lesion’s relationship to the levator ani muscle, obturator internus muscle, vagina and anal canal. However, episiotomy scar endometriosis remains rare and MRI is also crucial to exclude differential diagnoses such as granuloma, chronic inflammation, or anal fistula.

Pain management in patients with pudendal neuropathy includes medical treatment with drugs (antidepressants or neuromodulators) and perineural injection of corticosteroids and lidocaine or bupivacaine [55]. In most cases, these injections offer temporary symptom relief. Pudendal nerve decompression surgery may be considered for patients who do not respond to medical treatment.

Treatment of episiotomy scar endometriosis may require surgical excision to minimize the risk of recurrence. Recently, image-guided percutaneous treatments using cryotherapy have been considered [57].

### Lumbar plexus

The lumbar plexus is formed by the ventral rami of the L1–L4 nerve roots. It descends within or posterior to the psoas major muscle, anterior to the L2–L5 transverse processes, before exiting into the pelvis [45]. Key nerves arising from this plexus include the obturator nerve and the femoral.

#### Obturator nerve

The obturator nerve is formed by the ventral rami of L2–L4. It arises from the medial border of the psoas major muscle and descends along the pelvic brim. It joins the obturator vessels and passes through the superolateral aspect of the obturator foramen within the obturator canal [45]. The obturator nerve provides motor innervation to the adductor muscles of the hip and sensory innervation to the medial thigh and knee.

Although relatively small, this nerve is of sufficient caliber to be visualized on MRI, particularly using 3D T2W sequences. It is well depicted in its proximal course, medial to the psoas major at L5, and then follows a descending, anterior course to join the obturator vascular pedicle, which is easily identified by its characteristic flow void appearance (Supplemental Fig. 8).

Symptomatic obturator nerve endometriosis is rare and manifests with inner thigh pain, thigh adduction weakness, or difficulty in walking [58].

**Fig. 10:**
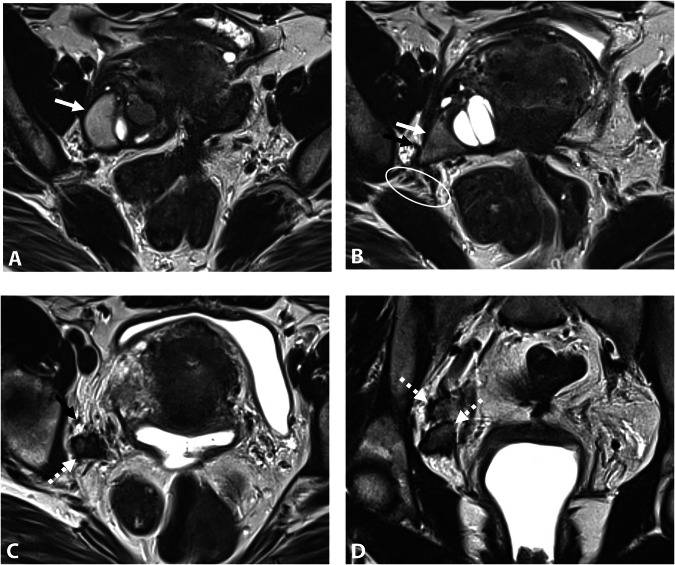
DE in a 29-year-old woman with deep dyspareunia, right sciatica, pain in the right inner thigh, and difficulty in walking. **A**–**C** Axial and (**D**) coronal T2W MR images show subperitoneal endometriosis lesion along and from the right adnexa (**A**, **B**, white arrows), extending to the right distal mediolateral parametrium (**C**, **D**, dashed arrows) and to the right obturator nerve (**B**, **C**, black arrows). The lesion is in contact with the right sciatic nerve posteriorly (**B**, circle), with an extrinsic traction effect but no involvement. After the failure of the medical treatment, the patient underwent surgery, including hysterectomy, right adnexectomy, right parametrectomy, ureterolysis, and dissection of the obturator nerve, which was entrapped by the mass

Endometriotic involvement of the obturator nerve is rare, it is commonly described in the obturator fossa [59–62]. It often results from the extension of a large endometriotic lesion from the ovarian fossa going through the mediolateral parametrium up to the obturator nerve (Fig. 10). MRI features include a retractile fibrotic infiltration with possible hemorrhagic cystic areas along the pelvic wall, tracking the course of the nerve. The MRI report should specify the relationship of the endometriosis lesion to the ureter and the obturator internus muscle. In chronic cases, nerve involvement may result in atrophy and fatty degeneration of the adductor muscles [59].

#### Femoral nerve

The femoral nerve—the largest branch of the lumbar plexus—is formed by the L2–L4 nerve roots. It arises from the lateral border of the psoas major muscle and runs inferolaterally between the psoas major and iliacus muscles (Supplemental Fig. 9), before exiting the pelvis beneath the inguinal ligament [45]. Functionally, the femoral nerve is responsible for hip flexion and knee extension and provides sensory innervation to the medial thigh, anteromedial knee, medial leg, and foot.

Femoral nerve endometriosis manifests with catamenial episodes of cruralgia and partial sensory and motor loss in the femoral nerve territory [23].

**Fig. 11:**
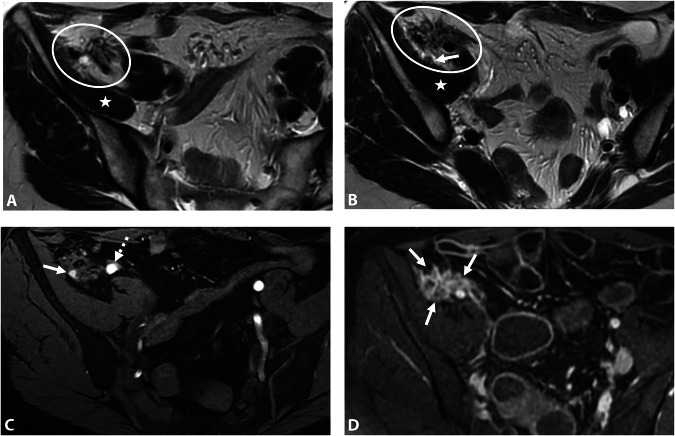
Femoral nerve endometriosis in a 34-year-old woman. **A**, **B** Axial 3D reconstruction T2W MR images show a fibrotic and retractile infiltration (circles) in contact with the right iliacus muscle (stars), on the course of the right femoral nerve (arrow). **C** Axial 3D reconstruction fat-suppressed T1W MR image reveals some T1-hyperintense endometriotic hemorrhagic microcysts (arrow), not to be confused with the external iliac artery (dashed arrow). **D** Axial T1W post-contrast MR image shows persistent enhancement of the lesion (arrows)

Nerve involvement in endometriosis is exceptional and generally results from a large endometriotic lesion extending along the course of the nerve, either in the ilio-psoas muscle region (Fig. 11) [23] or in the inguinal region following the distal third of the round ligament [63]. In these cases, the relationship to the inguinal canal and the common femoral artery and vein should be described.

## Conclusion

Pelvic nerve involvement in endometriosis is a condition often underestimated by practitioners and underreported by radiologists. Early diagnosis is essential to prevent irreversible nerve damage. MRI is the optimal imaging modality to accurately assess the distribution and the extent of pelvic nerve involvement in endometriosis. Recognizing the key features of MRI is essential for radiologists to provide structured reports to assist in selecting the best therapeutic approach and patient counseling.

## Supplementary information

ELECTRONIC SUPPLEMENTARY MATERIAL

## Data Availability

All patients’ clinical and radiological data were from Lyon Sud University Hospital and Tenon University Hospital.

## Abbreviations

3D: Three-dimensional
DE: Deep endometriosis
DTI: Diffusion tensor imaging
MRI: Magnetic resonance imaging
T1W: T1-weighted
T2W: T2-weighted
