# Supplementary material for: Pelvic nerve endometriosis: MRI features and key findings for surgical decision
Source: Insights Imaging. 2025 Jun 19;16:131. doi: 10.1186/s13244-025-02005-6 (PMC12179019; doi:10.1186/s13244-025-02005-6)

Pelvic Nerve Endometriosis: MRI features and key findings for surgical decision

ELECTRONIC SUPPLEMENTARY MATERIEL

Insights Imaging (2025) Bourg J, Ruaux E, Bolze PA, et al.

Figure 1 - Illustration of the mediolateral and posterolateral parametrium

The mediolateral parametrium is defined as the space lateral to the uterine cervix and vagina between an anterior line that is anterior to the cervix or vagina, and a posterior line that is anterior to the rectum (light orange area). The posterolateral parametrium containing the sacrorectal septum is defined as the space behind the posterior line passing anterior to the rectum, between the mesorectal fascia and the termination of internal iliac vessels (dark orange area).

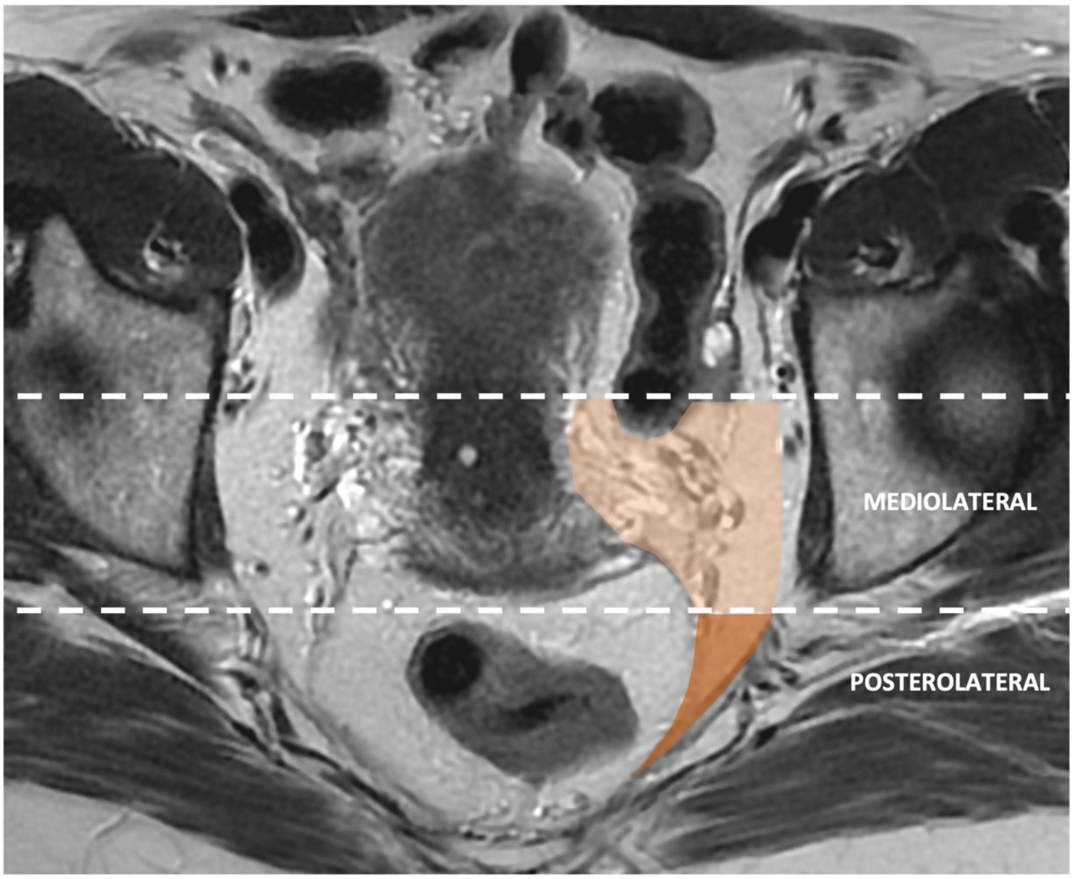

Figure 2 - Right inferior hypogastric plexus projection area in a woman with bladder endometriosis and internal adenomyosis in an obese patient

(a) Axial, (b) sagittal and (c) coronal 3DT2-weighted MR images show the right inferior hypogastric plexus projection area (orange dotted lines). The antero-inferior angle corresponds to the point of the contact of the ureter with the pelvis (white arrows). (d) The robotic laparoscopic view shows that after dissection of the roof of the right posterolateral parametrium, the right hypogastric nerve (arrows) enters the underlying hypogastric inferior plexus and lies outside the uterosacral ligament (white arrowheads) and under the ureter (black arrowheads). Note the pouch of Douglas (black star) and the anterior rectal wall (white stars)

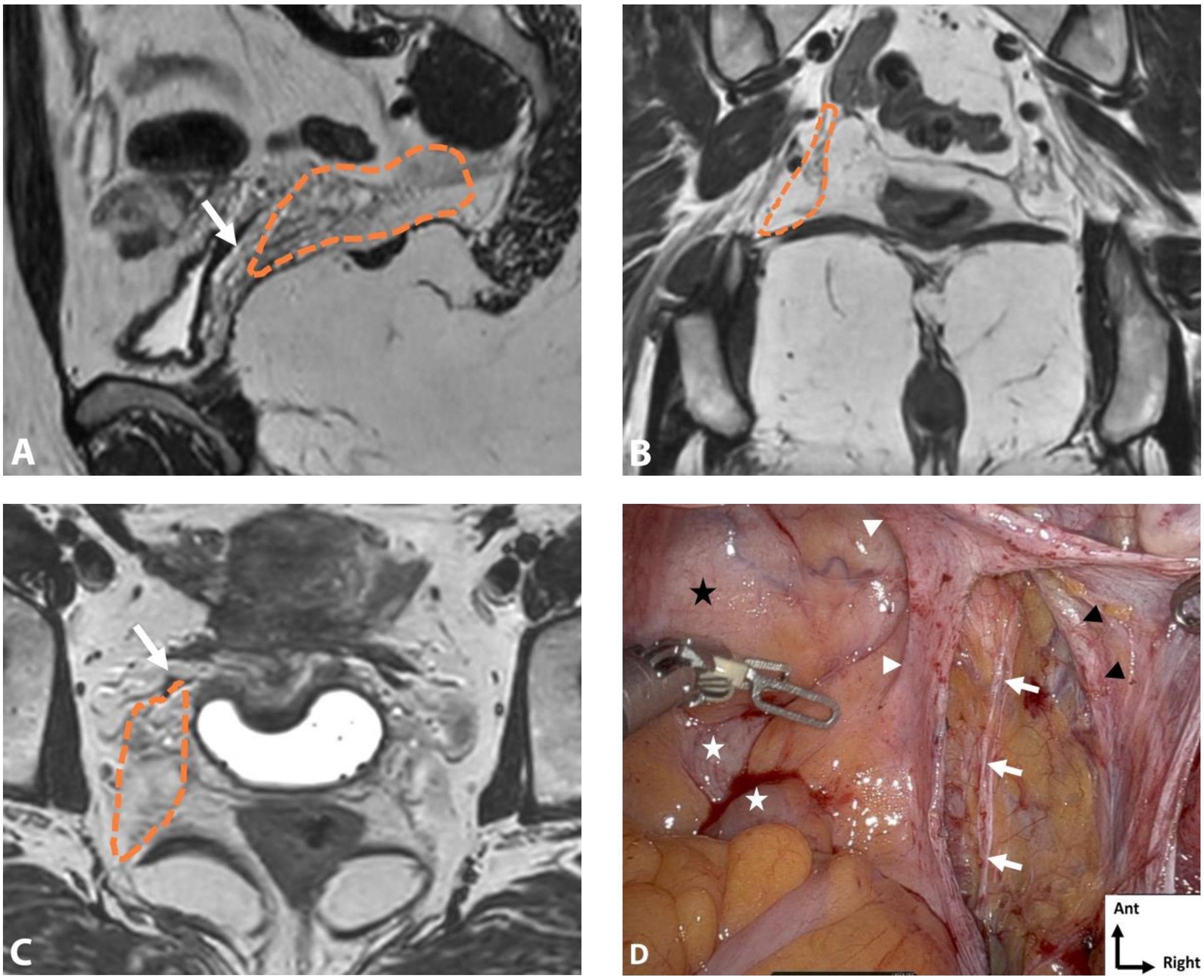

Figure 3 - Normal course of the sacral roots.

(a, b, c, d) Axial 3D T2-weighted MR images show the right sacral roots, well traced at the level of the foramen and more difficult to see in their course, with a decrease in caliber from S1 to S4.

Note the proximal course of S2 and S3 in the muscular indentations of the piriformis muscle (stars), which facilitates their visualization.

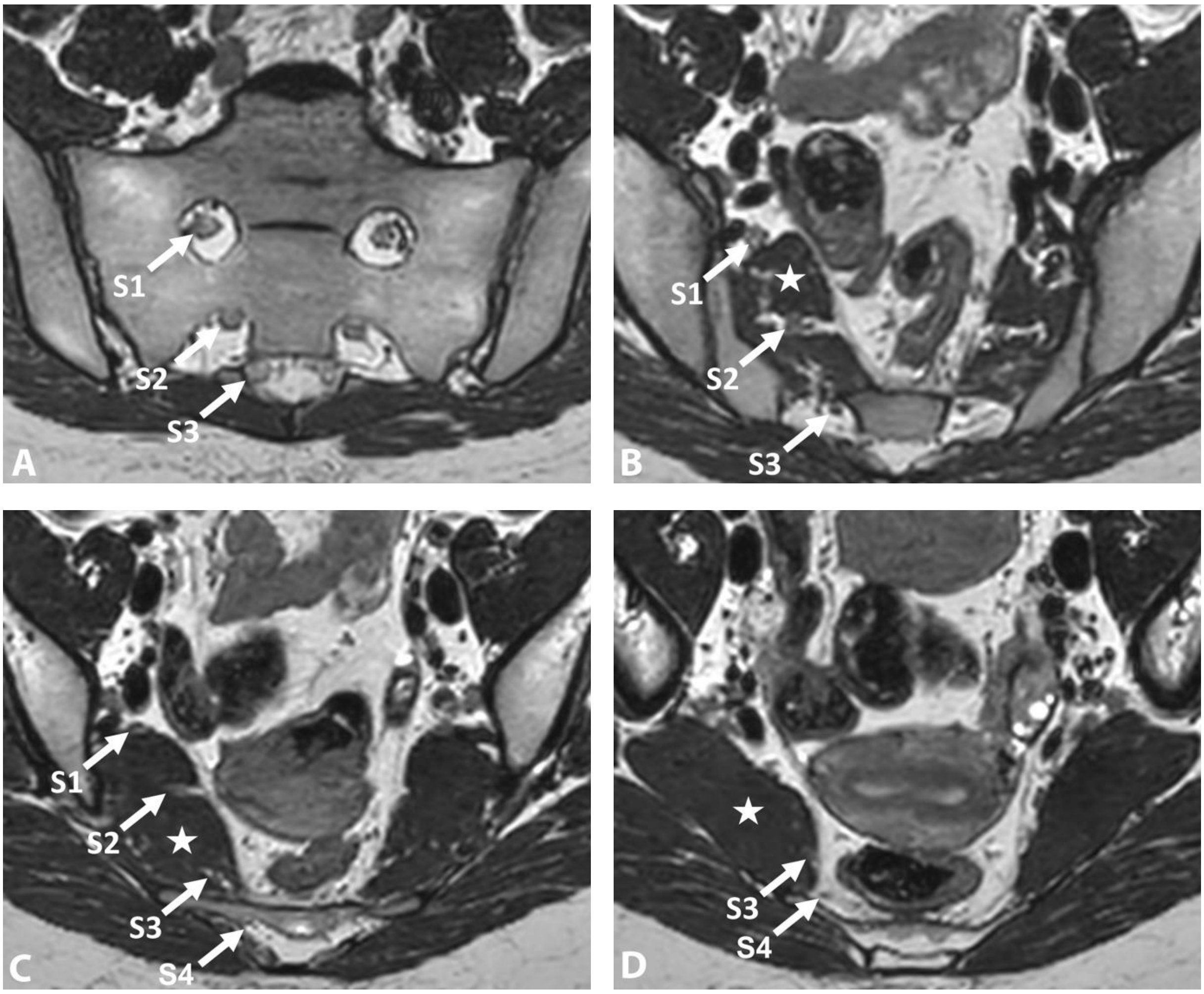

Figure 4 – Superior view dissection of the right sacral roots and obturator nerve (fresh cadaver).

The photograph shows the exposure of the right sacral roots and obturator nerve.

Note the resection of the external iliac vessels (star).

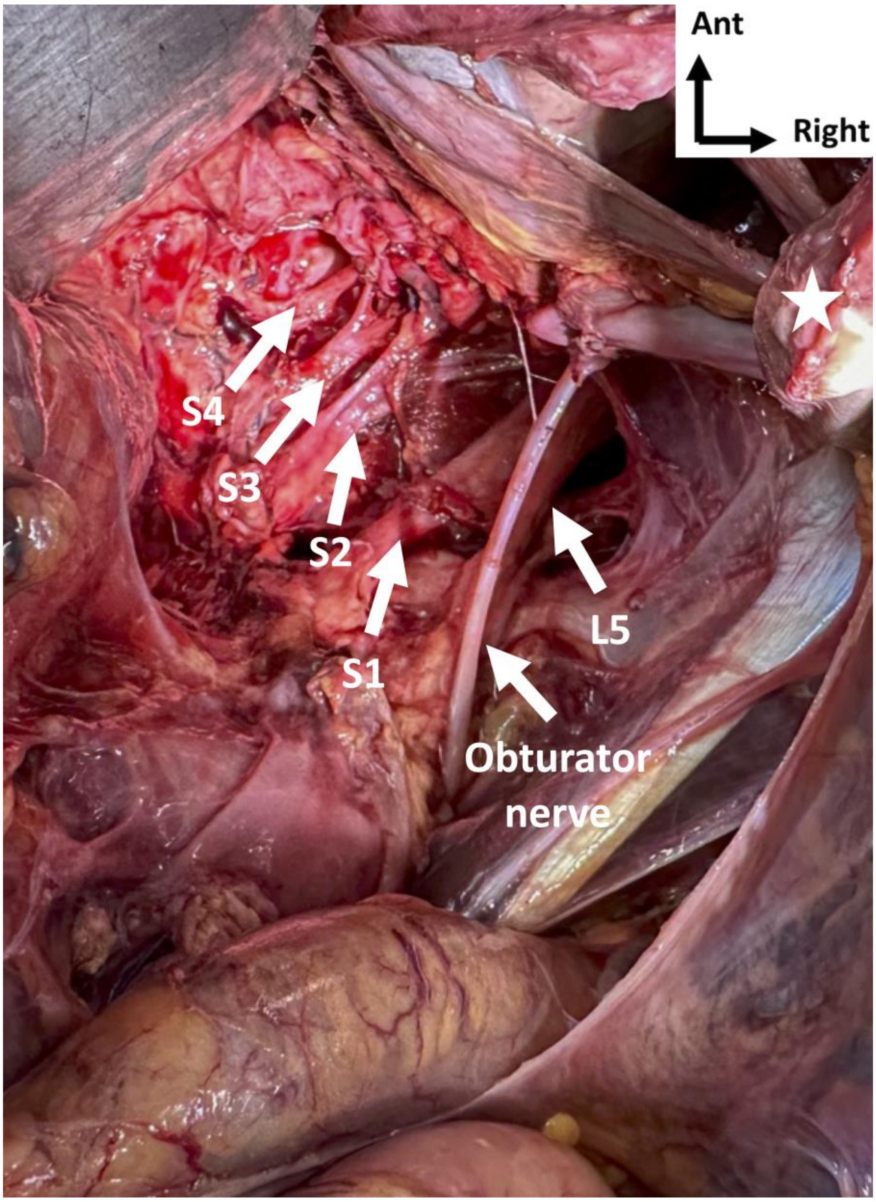

Figure 5 - Deep endometriosis in a 35-year-old woman with pain and dysesthesia in the right lower extremity.

(a) Axial and (b) sagittal 3D reconstruction T2-weighted MR images show fibrotic infiltration of the torus (A, arrow) extending to the right posterolateral parametrium with involvement of the inferior hypogastric plexus (stars) and spiculations extending posteriorly to the S2-S3 sacral nerve roots (dashed arrows).

(c, d) Axial and (e) sagittal 3D reconstruction fat-suppressed T1 MR images show hemorrhagic implants in the right posterolateral parametrium, and hemorrhagic thickening of the S2 and S3 sacral nerve roots (C-D, arrows) with retro-extension to their extraforaminal portions and up to the foramen for S3 (E, dashed arrow).

The patient was medically treated with an LHRH analogue.

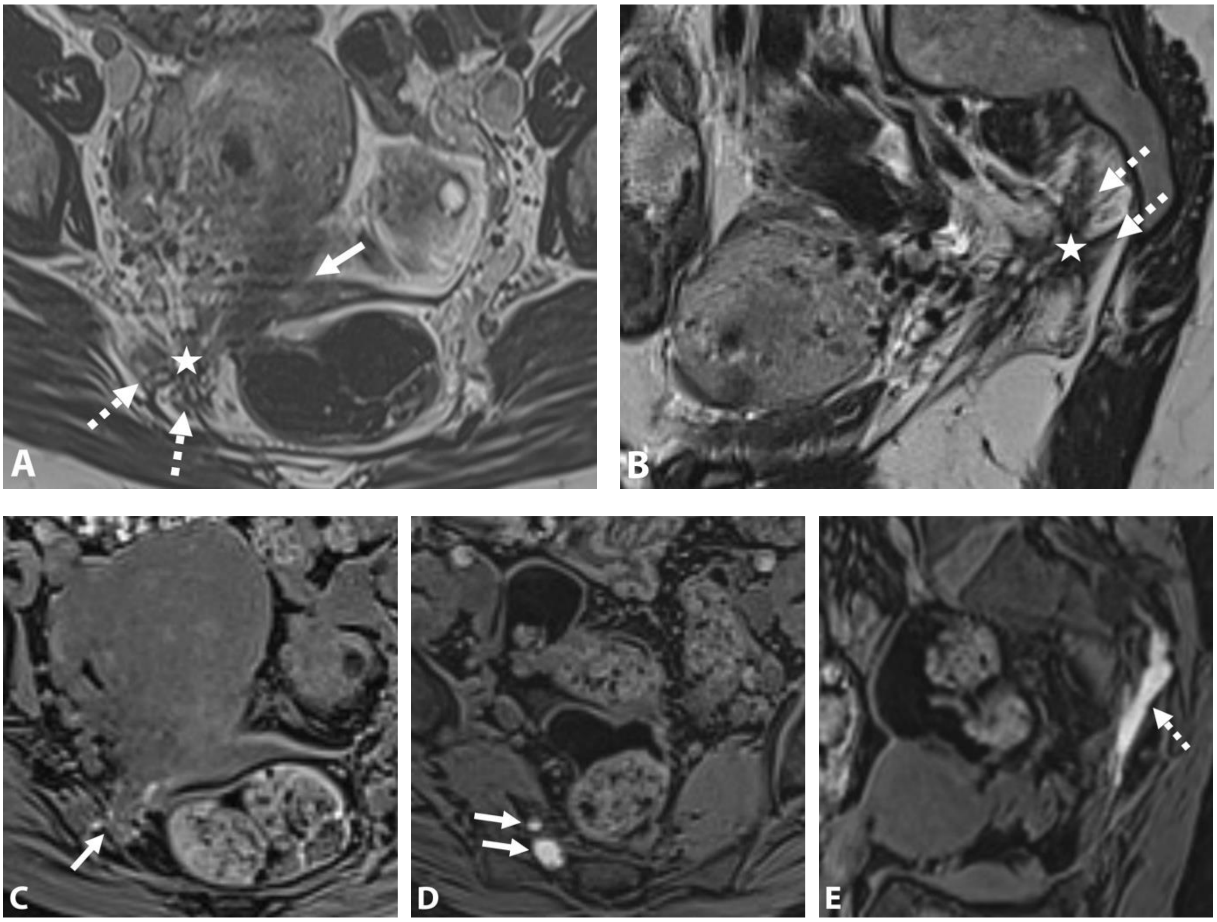

Figure 6 - Normal course of the sciatic nerve.

(a) Axial and (b) sagittal 3D T2-weighted MR images show the left sciatic nerve (oval) passing anterior to the piriformis muscle (stars) with a “spaghetti-like” aspect.

(c) Coronal oblique 3D reconstruction T2-weighted MR images show the left sciatic nerve (arrows) passing above and outside the ischial spine (dashed arrow).

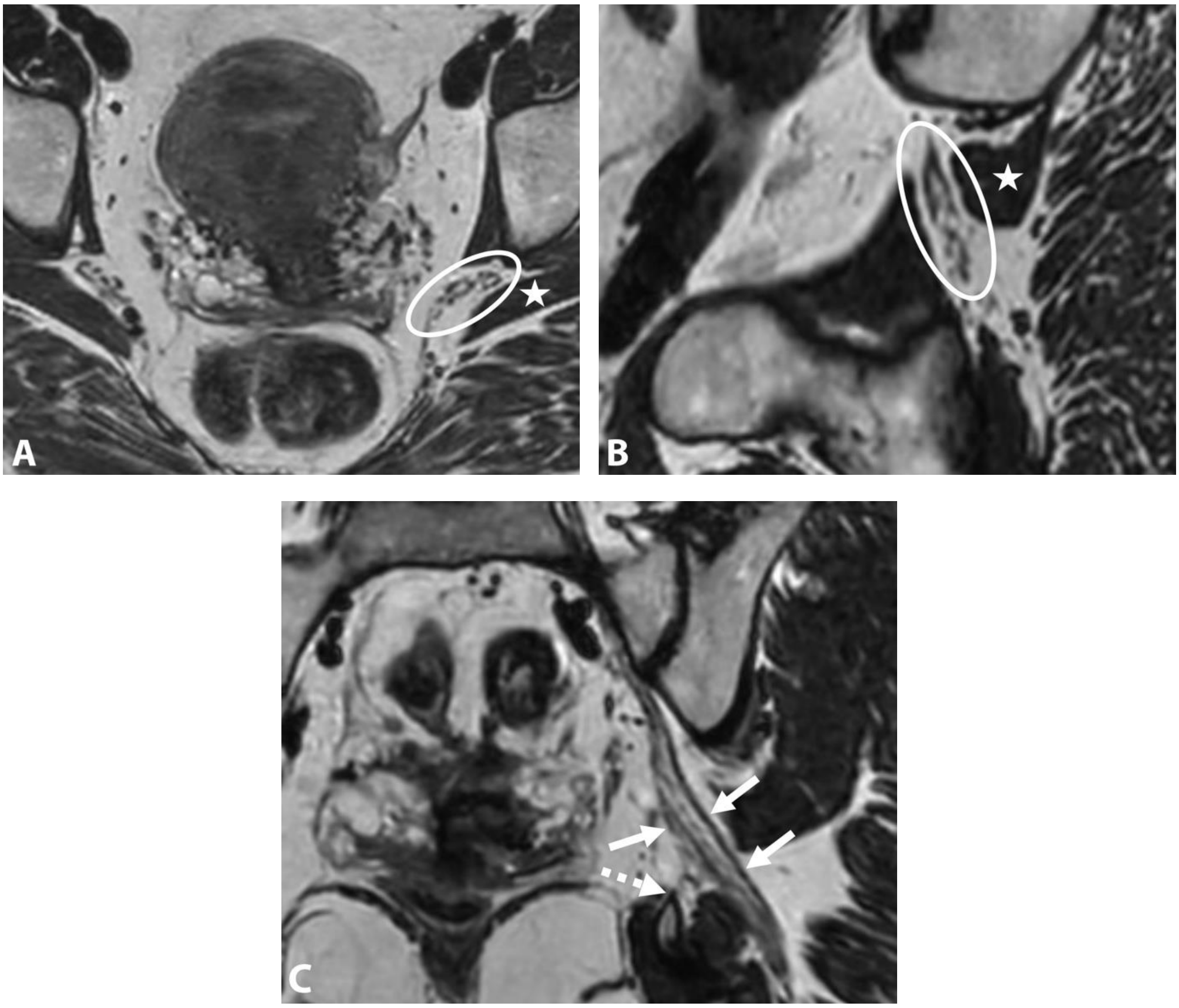

Figure 7 - Normal course of the pudendal nerve.

(a) Axial 3DT2-weighted MR image shows the theoretical area of coalescence of the S2-S4 nerve roots forming the left pudendal nerve (circle) at the junction in the middle third of the piriformis muscle (star).

(b) Axial 3DT2-weighted MR image shows the anatomic window of the left pudendal nerve course (circle) between the sacrospinous (black arrow) and sacrotuberous (white arrow) ligaments.

(c) Axial and (d) coronal 3DT2-weighted MR images show the left pudendal nerve and the internal pudendal vessels courses (circles) in the Alcock's canal, bounded by the fascia of the obturator internus muscle (stars).

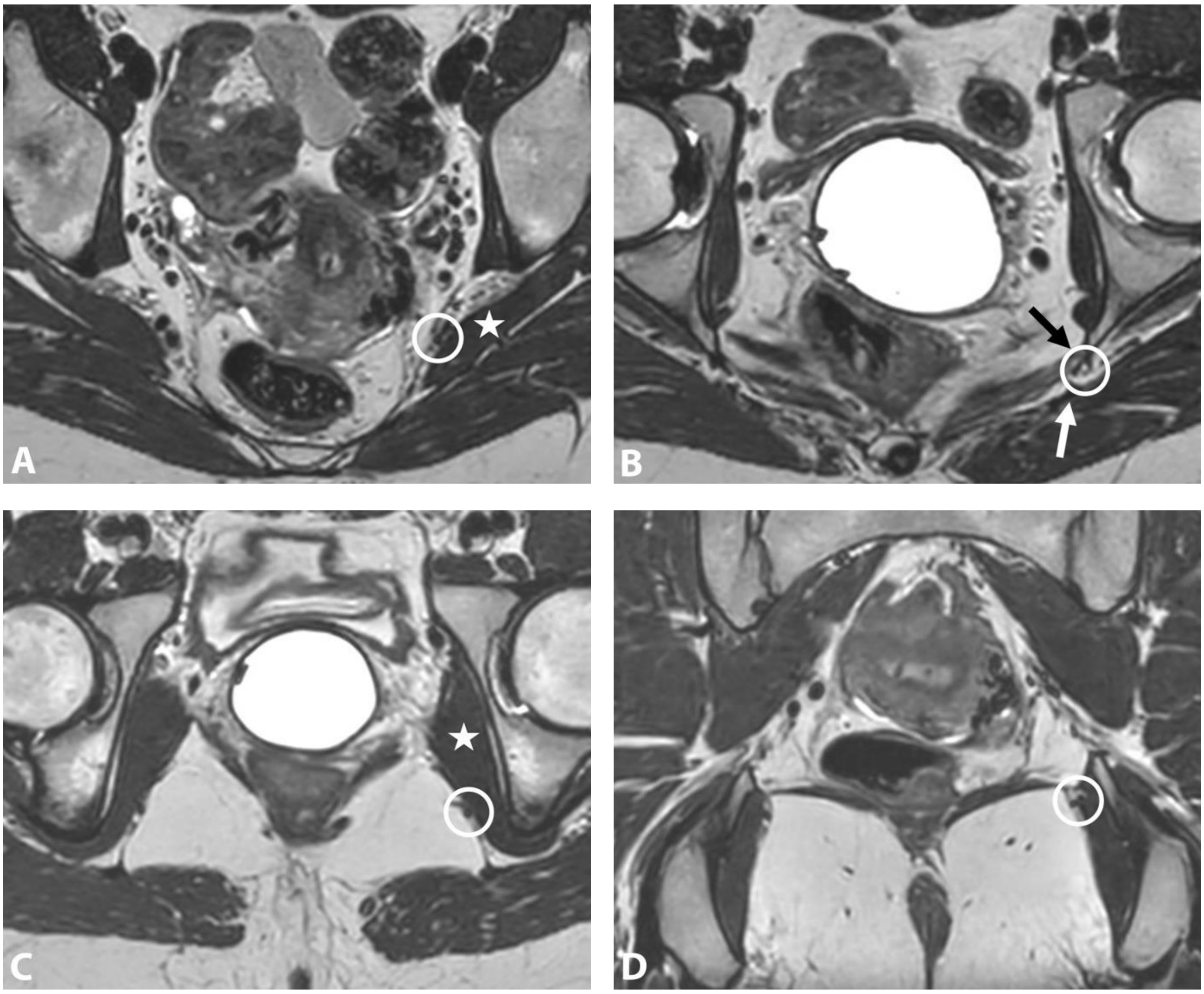

Figure 8 - Normal course of the obturator nerve

(a) Axial 3D T2-weighted MR image shows the right obturator nerve (arrow) descending medially and posteriorly to the psoas major muscle (star).

(b) Axial 3D T2-weighted MR image shows the right obturator nerve (arrow) passing along the pelvic brim in front of the obturator vessels (dashed arrow).

(c) Axial 3D T2-weighted MR image shows the right obturator nerve (black arrow) passing through the upper part of the obturator foramen with the obturator vessels, anterior to the obturator internus muscle (star).

(d) Sagittal oblique 3D reconstruction T2-weighted MR image shows the vertical course of the right obturator nerve (arrows).

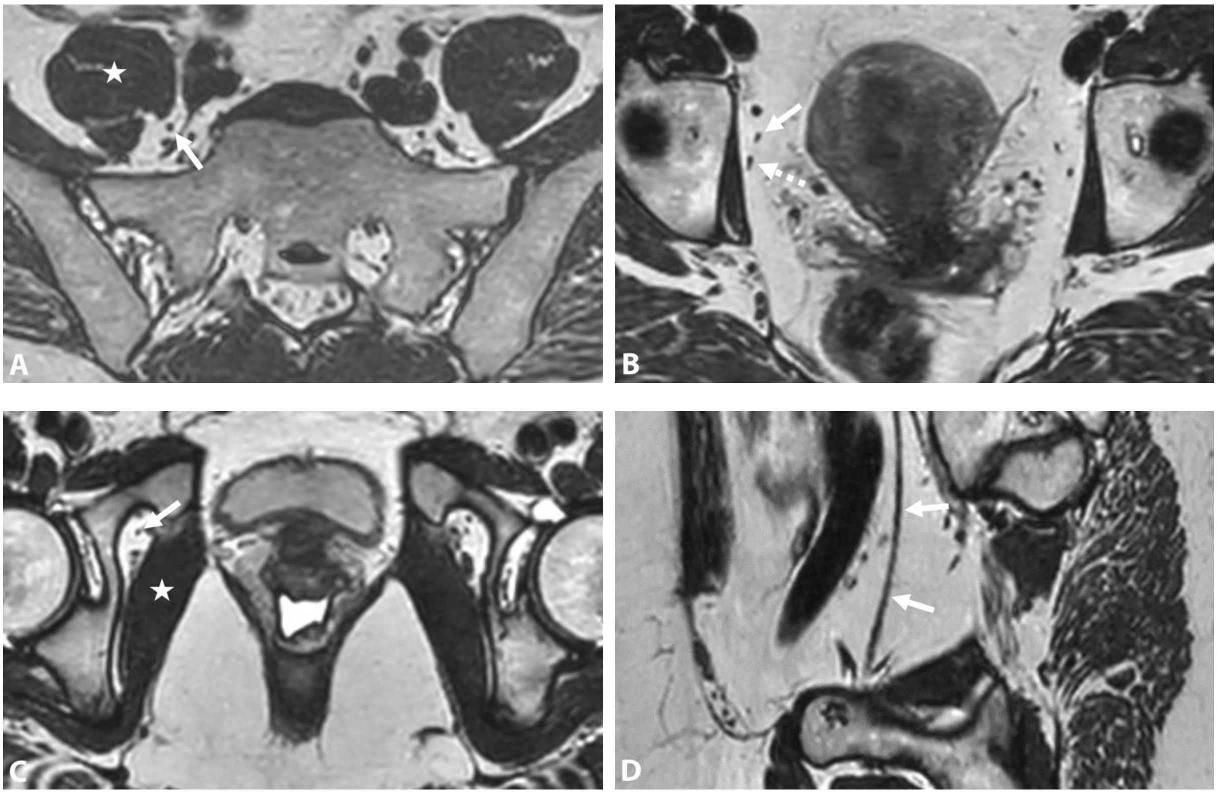

Figure 9 - Normal course of the femoral nerve.

(a) Axial T2-weighted MR image shows the right femoral nerve (arrow) ŧ arising from the lateral border of the psoas major muscle (star) and running laterally to it.

(b) Axial T2-weighted MR image at the level of the middle course of the round ligament of the uterus (dashed arrow) shows the right femoral nerve (arrow) running anterior to the iliacus muscle (star).

(c) Axial T2-weighted MR image shows the right femoral nerve (arrow) exiting the pelvis below and lateral to inguinal canal (circle).

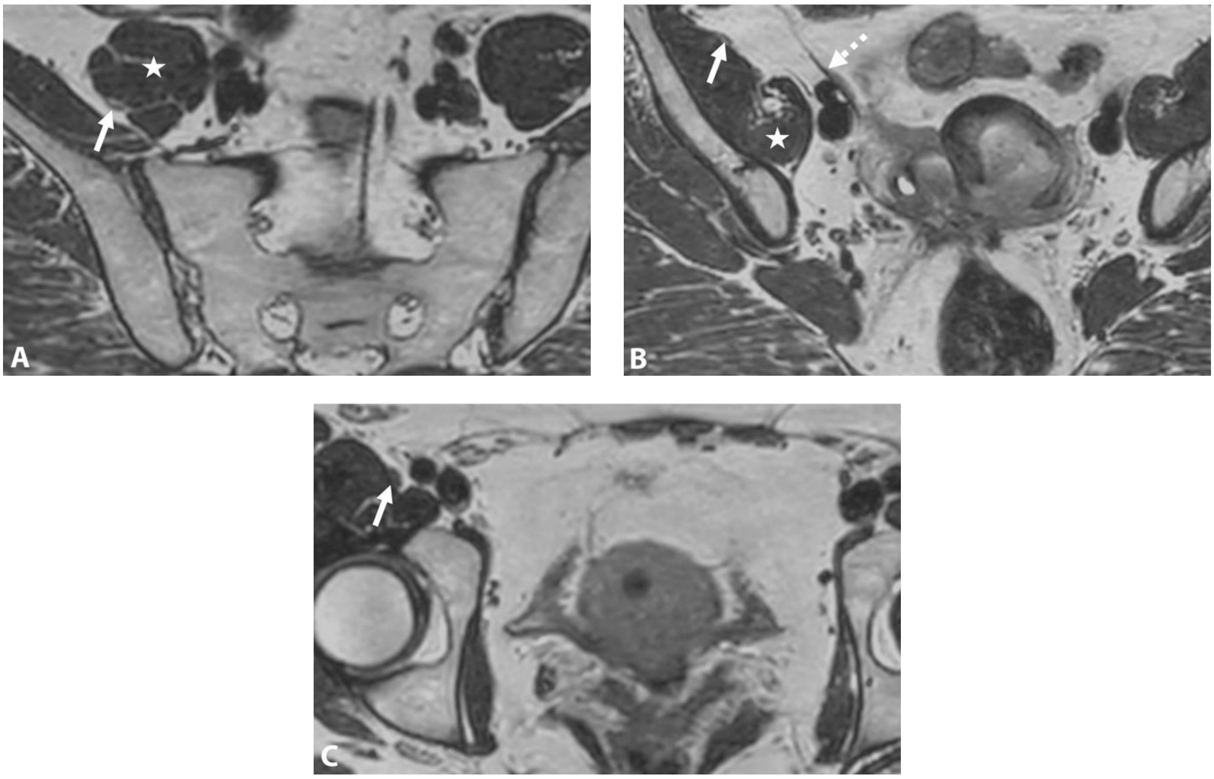

Supplement: Supplementary file 1 — ELECTRONIC SUPPLEMENTARY MATERIAL [file 13244_2025_2005_MOESM1_ESM.pdf]
